# Concepts of Mental Disorders Among Psychiatrists, Psychologists, and Theologians

**DOI:** 10.3390/ejihpe14110185

**Published:** 2024-10-25

**Authors:** Žana Kralj, Goran Kardum

**Affiliations:** 1 Clinic for Psychiatry, University Hospital of Split, 21 000 Split, Croatia; zkralj@kbsplit.hr; 2 University of Split School of Medicine, 21 000 Split, Croatia; 3 Department of Psychology, Faculty of Humanities and Social Sciences, University of Split, 21 000 Split, Croatia

**Keywords:** mental disorders, concepts, soul, mind, self

## Abstract

The objective of this study was to compare the perspectives of psychiatrists, psychologists, and theologians on schizophrenia, depression, anxiety, and antisocial personality disorder. A cross-sectional research design was utilized, involving a random sample selected from the official registries of these professionals. The findings revealed significant differences in how these groups conceptualize mental disorders. The preferred concepts varied depending on the specific disorder. For instance, when it came to schizophrenia, psychiatrists showed a greater inclination towards the psychodynamic concept compared to psychologists, who leaned more towards the cognitive and social constructivist concepts. In the case of depression, psychiatrists favored biological, psychodynamic, and spiritual concepts, while psychologists tended to prefer the cognitive concept. Theologians consistently favored the spiritual concept across all four diagnoses, in comparison to both psychiatrists and psychologists. This research holds significant value for both theoretical understanding and practical applications, and future studies should consider incorporating qualitative, in-depth research to explore the complexities of these concepts related to mental disorders.

## 1. Introduction

The research and treatment of mental disorders encompass a wide range of fields, including medicine, psychology, philosophy, and more. Mental health goes beyond medical issues. Psychiatry is that branch of the medical profession which deals with the origin, diagnosis, prevention, and management of mental disorders or mental illness, emotional, and behavioral disturbances [1]. In a healthcare institution, they use medication and different psychotherapeutic techniques to treat patients. Psychologists have a psychology degree and study human behavior, the mind, and various aspects of mental life. They can also apply different psychotherapeutic techniques. The field of clinical psychology is solely dedicated to studying and treating mental illnesses. In Croatia, theologians are scholars who dedicate their research to the systematic study of the divine, religious beliefs, and doctrines. Their usual responsibilities include leading worship, administering sacraments, preaching, teaching, and providing spiritual support to the community. Radden [2] states that philosophy deals with ethical inquiries about the mind–body relationship and individual identity. Sociology studies the influence of social factors on mental health, while anthropology explores cultural viewpoints on mental disorders [3,4]. Religion and spirituality provide support and coping strategies, while legal professionals focus on mental health issues within the legal system [5]. Furthermore, education plays a crucial role in fostering mental well-being.

The construct of mental health touches upon countless topics, such as the mind–body relationship, mental disorders, classification, etiology, treatment, and so on. It is important to note that mental health is not an isolated concept. It provides individuals with a sense of worth, control, and comprehension of their internal and external functioning [6]. Ref. [7] argues that taking an interdisciplinary approach to mental health treatment allows for a comprehensive understanding and effective collaboration. Insufficient understanding of concepts, values, and activities across professions or disciplines can hinder daily work. Mental health professionals, priests, and the general public are influenced by multiple models of mental illness, which shape their understanding of mental health.

### 1.1. Mental Health and Disorder

The understanding and acceptance of mental health concepts in psychiatry have changed throughout history. Psychiatry in the 21st century faces significant challenges, particularly in terms of validity [8]. Placing excessive emphasis on efficacy studies has introduced weaknesses and risks in the health field by disregarding the exploration of treatment theories and mechanisms [9]. Galderisi et al. [10] aims to move beyond ideal norms and theoretical traditions, favoring an inclusive approach that embraces diverse perspectives and reflects human life experience.

The cultural context also influences the definition of mental health. Wang [11] introduces a Yin–Yang definition model of mental health that incorporates Confucianism and Taoism theories of personality. This model accurately reflects traditional Chinese cultural beliefs about mental health and provides insights into East Asian psychotherapy and mental health practices within the Chinese cultural context. Yamada et al. [12] found that Korean pastors placed high importance on bad parenting and demon possession as causes of mental illness, whereas Euro-American pastors prioritized genetics and chemical imbalances.

The construction of nosological entities relies on descriptive psychopathology, as observed in the works of Kraepelin, Jaspers, and Kurt Schneider. This pseudo-theoretical presentation may have influenced some inexperienced clinicians to view the DSM criteria as unquestionable and unchanging, hindering them from exploring alternative research avenues [8]. The current DSM-5 was influenced by a tradition marked by questionable science and politically motivated choices. Kelly et al. [13] marks the 50th anniversary of *The Myth of Mental Illness* by providing commentaries on its contemporary relevance from the perspectives of a range of stakeholders, including a consultant psychiatrist, psychiatric patient, professor of philosophy and mental health, a specialist registrar in psychiatry, and a lecturer in psychiatry. Psychiatric disorders have been found to have a complex genetic architecture with multiple risk alleles, based on studies conducted over the past decade. GWASs (Genome Wide Association Studies) have identified 108 schizophrenia-associated loci and 14 bipolar disorder-associated loci [14]. The Diagnostic Interview for Genetic Studies (DIGS) was created in 1994 by collaborators at the National Institute for Mental Health Genetic Initiative and has been translated into various languages [15]. The study conducted by [16] revealed the exceptional validity and reliability of the Croatian DIGS version, greatly improving the feasibility of genetic research on psychiatric disorders in the region. Therapeutic needs have been further clarified. Ref. [17] emphasizes the need for consistent treatment for patients with schizophrenia and the high likelihood of symptomatic relapse if medication is stopped, which is prevalent among this patient population.

### 1.2. The Intersection of Theory, Science, and Practice

The correlation between theory, science, and practice is peculiar and at times paradoxical. First and foremost, the mind–body relationship remains an unsolved problem, along with the complex connections between biology, social science, anthropology, philosophy, and more [18]. Where do we stand? It is suggested by Starmans and Bloom [19] that both children and adults have an intuitive sense that the self is positioned within the body, specifically at or close to the eyes. Limanowski and Hecht [20] point out that, throughout history, distinct bodily organs, like the heart (Aristotle) or the pineal gland (Descartes) have been proposed as the “seat” of the self (or soul). According to [21], Descartes’ theory regarding the soul and the pineal gland is more innovative than commonly thought. The core of Cartesian dualistic philosophy lies in the problem of the substantial union of the soul and body, and their mechanisms of interaction. The pineal gland’s central location and unique shape have sparked various metaphysical theories about its function. However, René Descartes’ philosophy suggests that its most original role is as the seat of the soul, where the interaction between the soul and body occurs [22]. The location of the human soul has been a contentious topic across philosophy, theology, and science [23].

The concept of mind in history reveals that humans possess both a physical body and a psychological capacity. The term “body” refers to the physical characteristics of a person, similar to how “mind” refers to their psychological attributes. The place where my body is located is where I am situated. Is there a specific location within my body where I exist? Recent results have provided contradictory findings about this question. According to [24], two different areas, the upper face and upper torso, are perceived as the location of “I”.

Modern philosophers often place the self within the human brain, which appears to have a significant influence. However, the concepts in today’s society create opportunities for ideological and political debates. In modernity, cerebral subject and brainhood serve as self identities and anthropological figures [25]. The interplay between the mind and body has been a subject of debate across different disciplines like science, philosophy, and religion for thousands of years.

The mind–brain problem has been inadequately addressed in the psychiatric literature, and a systematic review revealed a lack of proper presentation and discussion of the mind–body problem. Moreira-Almeida et al. [26] demonstrated that the limited articles on the subject were heavily cited, but exposed misrepresentations and a lack of thorough philosophical discourse, along with a pronounced bias against dualism and in favor of a materialist/physicalist approach to psychiatry. The psychiatric literature in some areas demonstrates a strong opposition to dualism. However, this opposition fails to accurately represent dualism as it conflates two mental concepts and disregards the existence of diverse dualistic perspectives [27]. Dualistic attitudes were most accurately predicted by religious belief. Even though most healthcare workers disagreed on the separation of consciousness and the physical body, more than one-third of medical and paramedical professionals saw the mind and brain as separate entities [28]. Religious belief and mental concepts about the relationship between mind and body obviously play an important role in concepts and models about mental health, disorders, treatments, and etiology. The book *Descartes’ Error* [29] underlined the significance of emotions and the body in thinking and decision making, arguing for the inseparable connection between the mind and body. By studying emotions and somatic markers, his research proves that our bodily feelings greatly affect our cognitive functions, questioning the mind–body separation.

### 1.3. Exploring the Concepts of Mental Disorders

Richter and Dixon [30] conducted a quasi-systematic review of theoretical models of mental health problems, analyzing 110 publications. They categorized 34 different models into five broader categories, with biological and psychological approaches being the most prevalent, and social, consumer, and cultural models being less diverse. In the field of mental health and psychotherapy, various approaches exist and many questions remain unanswered. Wagenfeld-Heintz [31] found out that most of the study participants were found to believe that medical–scientific and religious paradigms are equally important and may coexist or even be integrated in psychotherapeutic practice. Studies indicate that spirituality has a protective effect on mental health, reducing the risk of psychopathology [32,33]. Lower levels of depression, anxiety, and stress are connected to spiritual practices such as meditation, prayer, and active involvement in religious communities [34]. People who have a strong spirituality tend to have better coping skills, a stronger sense of purpose, and greater resilience, leading to improved emotional stability and a lower likelihood of mental disorders.

According to the biomedical model, mental disorders are viewed as brain diseases and treated with medication to address biological abnormalities. Deacon [35] stated that a biologically focused approach to science, policy, and practice has dominated the American healthcare system for more than three decades whereas the neglected biopsychosocial model represents an appealing alternative to the biomedical approach, and an honest and public dialog about the validity and utility of the biomedical paradigm is urgently needed.

The focus of psychological theories is on mental faculties such as perception, belief, emotion, and will, and the intelligent behavior they generate. These become the terms for describing psychological dysfunction, for construing the phenomena as “psychological dysfunction”—as opposed to for example sin, or expression of a disease [36].

The biopsychosocial model offers a contemporary, humanistic, and holistic perspective on human beings. George L. Engel (1913–1999), a prominent scholar in the psychosomatic movement, brought the model into the field of medicine. A growing body of research investigates the possibility of consciousness existing independently from the body [37].

The biopsychosocial model serves as both a clinical care philosophy and a practical guide. It offers a philosophical perspective on the interconnectedness between suffering, disease, and illness at different levels of organization, ranging from the societal to the molecular. From a practical standpoint, it allows us to acknowledge the patient’s subjective experience as a vital element in achieving accurate diagnosis, health outcomes, and empathetic care [38].

Research suggests that psychiatrists do not share a uniform perspective on mental disorders. While trainee psychiatrists prioritize the biological model for schizophrenia, they do not limit themselves to a single model. As a group, they organize their attitudes towards mental illness in terms of a biological/non-biological contrast, an “eclectic” view, and a psychodynamic/sociological contrast [39].

The general public usually agrees with evidence-based ideas about common disorders, but they may not always comprehend or accept how mental health professionals perceive them [40].

Although there are no disparities between laypeople and psychiatrists, differences do exist among mental health professionals. The findings suggest that psychologists and psychiatrists still have divergent perspectives on the biological–psychosocial continuum. Nonetheless, the study [41] pointed to a shared agreement on psychotherapeutic models. Clinical psychologists were more accepting of the antimedical paradigm compared to psychiatrists, psychiatric social workers, psychiatric nurses, and psychiatric outpatients. The medical model of mental disorders, as outlined by Blaney [42], has four significant implications: (a) mental disorders are organic diseases, (b) visible symptoms reflect underlying dysfunctions, (c) individuals are not responsible for their behavior, and (d) psychiatric symptoms are best understood through diagnostic procedures. These implications can be accepted or rejected selectively because they are independent. In any case, all show some level of support for the medical perspective. The medical model faces opposition from alternative models like psychological, behavioral, or moral approaches. The reasons why clinical psychologists seem to be leading the way in adopting a critical, antimedical approach in the clinical field are numerous [43]. Morrison and Hanson’s findings suggest that psychologists are taking the lead in moving away from the medical model of mental illness, partly to challenge the dominant position of psychiatrists in the mental health system.

According to Heseltine-Carp and Hoskins [44], Christian clergy are frequently seen as frontline mental health workers and key figures in mental health services. Unfortunately, there is still a lack of collaboration between clergy and mental health services. Clergy generally do not receive referrals from mental health professionals, despite the proven benefits. Previous studies indicate limited cooperation between clergy and professional mental health practitioners [45]. Individuals often seek guidance from clergy when facing emotional and mental health problems, yet mental health professionals do not often recognize clergy as valuable partners in mental healthcare [46]. Various historical factors, including ideological conflict, differing goals, and general distrust, have led to the clergy’s contributions to mental healthcare being undervalued.

The evidence shows that collaboration has multiple benefits, such as more referrals to mental health professionals, increased use of formal healthcare services, better treatment adherence, improved outcomes for mental disorders, and reduced stigma [47]. Recent research indicates a growing awareness of the connection between religion, spirituality, and mental health, and the importance of cooperation between religious leaders and mental health experts [48]. The study conducted by Aračić and Džinić focused on the views of Catholic priests, nuns, and citizens in Croatia about the support priests can offer to people facing daily challenges. The findings indicate that priests feel they can offer assistance in religious crises (91.9%), marital difficulties (75.0%), serious illness (70.6%), child-rearing (65.0%), and addiction (58.8%) [49].

Throughout history, the Church has been linked to the care of the sick. According to research conducted abroad, individuals suffering from mental health issues frequently seek guidance from clergy members before or instead of consulting healthcare professionals [46]. Secular journals emphasized the importance of education and knowledge more frequently than religious journals. Many viewed insufficient knowledge or education as a barrier to collaboration [45]. Religious advisors are crucial in mental healthcare and need proper training and collaboration with formal mental healthcare systems. Religious attitudes can be reliable indicators of how likely someone is to use religious advisors [50].

Religious themes were found to have a positive association with coping, treatment engagement, and help-seeking behavior. The results also suggest that family members and caregivers tend to prefer religious-based professionals and are cautious about mental health professionals. By studying the social support aspect, researchers and professionals can identify ways to enhance treatment by exploring the connection between religion and schizophrenia [51]. The World Mental Health Survey revealed that 12.3% of people receiving psychiatric care had previously sought assistance from a religious community representative [50]. As an illustration, literature from the US and UK often documents frequent consultations with clergy about emotional and psychological issues [52].

Religion plays a crucial role in the lives of schizophrenic patients, regardless of their delusional content. Despite being comfortable discussing the matter, clinicians were often unaware of their patients’ religious involvement [53].

The older priests had a higher level of conviction than the younger priests when it came to the efficacy of prayer and long-term pharmacotherapy. Priests need to be educated about the stigma surrounding mental disorders, especially schizophrenia [54]. Psychotherapy training, supervision, case discussions, and Balint groups are important tools highlighted by Őri et al. [55] to help psychiatrists combat the stigmatization of patients. Ref. [56] states that self-stigma and subjective medication side effect perception represent a relevant issue in patients’ life and should be carefully taken into account in clinical practice.

The objective of this study is to explore how psychiatrists, psychologists, and theologians conceptualize mental disorders.

## 2. Methodology

### 2.1. Participants and Setting

For the purposes of the research, a cross-sectional survey was conducted on psychiatrists (n = 121), psychologists (n = 116), and theologians (n = 74) in the Republic of Croatia who are members of the Croatian Psychiatric Association, the Croatian Psychological Chamber, and the Croatian Bishops’ Conference. Data collection for the first phase occurred between November and December 2017 [57], while the second phase spanned from 15 January to 15 June 2018 to reach a total of 311 participants. Psychiatrists and psychologists were professional staff from clinical hospitals, some of them worked both inpatient and outpatient, in public and private health clinics. The Psychiatric Society has a total of 300 members, which is equal to the number of psychologists in the Croatian Psychological Chamber with the status of health professionals. According to available data from the Croatian Bishops’ Conference from 2017, there are 2402 priests working in Croatia.

Participants took part in the study by accessing an online questionnaire through a provided link sent via email to relevant associations. The research questionnaires were made with the Google Forms app. The research procedures followed ethical standards set by the Declaration of Helsinki and were approved by the Ethics Committee of the Clinical Hospital Center Split. The survey could be completed voluntarily and participants were not compensated. All relevant information about the parts of the survey is listed at the beginning of the questionnaire and in the accompanying message describing the purpose of the research in general, indicating that the research is anonymous and that the confidentiality of the answers is ensured.

Participants were told that completing the entire survey and confirming their responses constituted informed consent. The Google Forms app was set up to ensure respondent privacy by not collecting personal data or asking identifying questions.

We randomly picked 150 email addresses from each group of respondents who are members of the Croatian Psychiatric Association, the Croatian Psychological Chamber, and the Croatian Bishops’ Conference based on the previous sample size calculation. Participants were contacted via email 3 times, with each message sent around 2 months apart. A total of 450 questionnaires were distributed, with 327 being returned. The response rate, overall, reached 73%. Nevertheless, data from 15 participants were excluded due to incomplete or incorrect questionnaires. The study had a total of 312 participants.

### 2.2. Measures

Attitudes of psychiatrists, psychologists, and theologians towards mental disorders were examined using the MAQ questionnaire, developed by Harland et al. [39]. The survey includes 32 statements rated on a Likert scale from 1 to 5, measuring attitudes towards four DSM diagnostic categories: schizophrenia, major depressive disorder, generalized anxiety disorder, and antisocial personality disorder.

In the DSM-IV guidelines [58], the American Psychiatric Association outlined four diagnoses. Schizophrenia is a chronic and severe mental disorder that has a profound impact on an individual’s thoughts, emotions, and behaviors. This condition is characterized by the presence of two or more of these symptoms for a significant duration of one month (or less with effective treatment): delusions, hallucinations, disorganized speech, grossly disorganized or catatonic behavior, and negative symptoms.

The DSM-IV defines major depressive disorder as the occurrence of one or more major depressive episodes. For a major depressive episode to be diagnosed, it is required that five or more of these symptoms occur continuously for two weeks. This indicates a shift from previous functioning, with at least one symptom being either a feeling of sadness or a decrease in interest or pleasure, depressed mood most of the day nearly every day, markedly diminished interest or pleasure in almost all activities, significant weight loss when not dieting or weight gain, changes in appetite, insomnia or hypersomnia, psychomotor agitation or retardation, fatigue or loss of energy, feelings of worthlessness or excessive guilt, diminished ability to think or concentrate, and recurrent thoughts of death or suicidal ideation.

Generalized anxiety disorder is a condition where individuals experience excessive anxiety and worry for at least six months about different events or activities. Three or more of the following symptoms must be present to associate anxiety and worry: restlessness or feeling keyed up, easy fatigue, difficulty concentrating or a blank mind, irritability, muscle tension, and sleep disturbances, such as difficulty falling or staying asleep, or experiencing restless and unsatisfying sleep.

Antisocial personality disorder is a condition where individuals consistently disregard and violate the rights of others since age 15, as shown by three or more criteria; failure to conform to social norms regarding lawful behaviors, deceitfulness demonstrated through repeated lying, use of aliases, or conning others for personal gain, impulsivity or a failure to plan, irritability and aggressiveness shown by repeated physical fights or assaults, reckless disregard for the safety of oneself or others, consistent irresponsibility reflected in repeated failures to maintain steady work or honor financial obligations, and a lack of remorse, evident in indifference or rationalization of having harmed, mistreated, or stolen from others.

The MAQ items and total score represent eight conceptual paradigms, namely biological, behavioral, cognitive, psychodynamic, social constructivism, social realism, spiritual, and nihilistic. The dimensions of mental disorders include etiology, classification, research, and treatment. Each model has a statement for each dimension, making a questionnaire of 32 randomly distributed statements (8 models × 4 dimensions). Participants need to evaluate their level of agreement or disagreement with each statement about a specific diagnostic category (statement [32] × diagnostic category [4]). In total, the questionnaire has 128 statements, which are divided into 4 disorders with 32 statements each.

Harland et al. [39] constructed a questionnaire and verified it using eight models. That model is supported by Read et al. [41], which reveals variations between psychologists and psychiatrists in those eight models. What characteristics distinguish each model and align with the MAQ questionnaire statements? Mental disorders are seen as brain diseases in the Biological Model, affected by genetics, neurochemicals, and physiology. Treatment mainly involves medications such as antidepressants and antipsychotics. The Cognitive Model emphasizes the significance of cognitive distortions and thought processes, tackling negative thinking patterns with cognitive–behavioral therapy (CBT). The Behavioral Model concentrates on observable behaviors acquired through conditioning, employing methods such as exposure therapy for reinforcement-based treatment. The Psychodynamic Model delves into unconscious processes and early life experiences, using psychoanalysis to uncover unresolved conflicts and repressed memories for greater understanding. The Social Realist Model explores the influence of social factors, such as socioeconomic status, and promotes interventions focused on the community. According to the Social Constructivist Model, mental health is influenced by social interactions and cultural contexts. Narrative therapy is used to change societal perceptions. The Nihilist Model adopts a skeptical position towards psychiatric practices, offering criticism of traditional diagnoses while not advocating for specific treatments. The role of spirituality in mental health is highlighted in the Spiritualist Model, which employs integrative methods to address issues like lack of spiritual fulfillment and existential crises.

### 2.3. Data Analysis

The data analysis was conducted using R software ver. 4.4.0 [59]. Before conducting the research, we estimated the sample size using the pwr and pwrss packages in the R environment [60,61]. To determine the sample size, we utilized average values, standard deviations, and effect sizes from prior studies involving psychiatrists and psychologists who completed the MAQ questionnaire [39,41]. Existing research in the field of mental health has failed to include three specific groups of experts and collaborators. To calculate the power and sample size, we made estimations for the values of the third group. We determined the sample size using a power of 0.85 and an alpha of 0.01, which is considered acceptable for behavioral sciences research [62]. Based on the mentioned input parameters, we need a sample size of approximately 100 respondents, which requires a group size greater than 33.

Descriptive statistics, such as averages, standard deviations, ranges, and total values, are presented before using inferential statistics procedures. The Shapiro–Wilk test was used to check the normality of the distribution.

We compared the preference for an implicit model of MAQ questionnaire among psychiatrists, psychologists, and theologians using multinomial regression analysis. Multinomial logistic regression is an attractive and appropriate analysis technique, as it does not rely on the assumption of normality. We utilized the multinom function from the nnet package ver. 7.3-19 [63]. The figures were created by utilizing the functions available in the ggplot2 package [64]. We set the significance level at 95% for multiple tests.

## 3. Results

Table 1 provides an overview of psychiatrists, psychologists, and theologians. The majority of psychiatrists and psychologists are women, while the Catholic Church’s theology field is predominantly male. Among psychologists, over 90% are women, while, in the field of psychiatry, 64% are female. There is also a slight difference in the age composition of these three samples. Within the field of psychiatry, just over 35% are aged between 46 and 55, whereas the majority of psychologists, specifically 45%, fall within the 26 to 35 age group. The age groups with the largest concentration of theologians are 36–45 (30%) and 56–65 (31%).

Psychologists have the lowest religious affiliation, with only 65 (56%) identifying as Catholics, compared to psychiatrists, where 90 (80%) identify as Catholics. The percentage of atheists is highest among psychologists, with 26 of them, or 22%, compared to only 4.6% among psychiatrists.

Table 2 presents the descriptive statistics for aggregate attitude scores, categorized by model and disorder. The Shapiro–Wilk test reveals significant deviations (*p* < 0.01) from the normal distribution for the mentioned concepts. Yet, we uncover more meaningful indicators when we conduct multinomial regression analysis.

**Figure 1:**
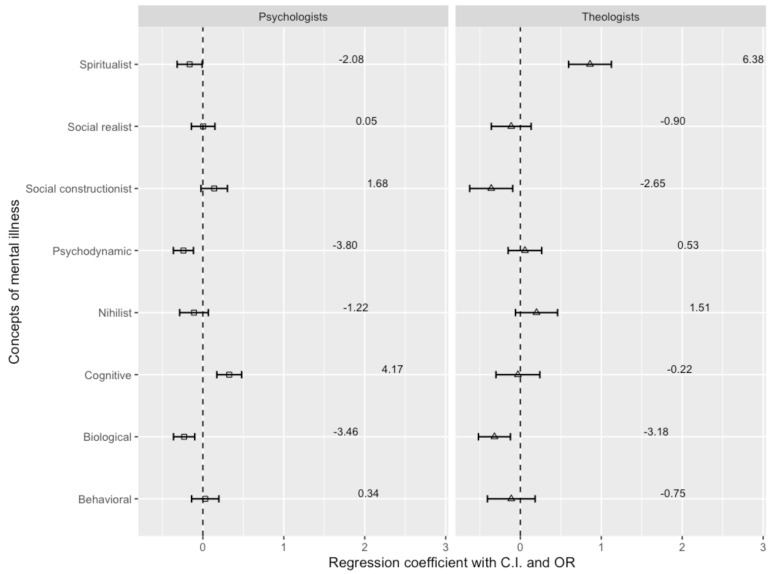
Psychiatrists’, psychologists’, and theologians’ concepts of major depressive disorder (psychiatrists as a reference group).

Figure 1 provides a visualization of the multinomial logistic regression major findings for depression, the estimated logistic regression coefficients, standard error bars, and corresponding odds ratios.

Compared to psychiatrists as a reference group, when it comes to major depressive disorder, psychologists are three times less likely to represent the biological (OR = −3.46, β = −0.232, *p* = 0.001) and psychodynamic (OR = −3.80, β = −0.240; *p* < 0.001) concept and twice less likely to represent the spiritual concept (OR = −2.06, β = −0.163, *p* = 0.038).The likelihood of psychologists endorsing the cognitive model is more than four times higher than that of psychiatrists (OR = 4.17, β = 0.326, *p* < 0.001). 

The likelihood of theologians supporting the spiritual concept for major depressive disorder is six times higher than that of psychiatrists (OR = 6.3, β = 0.861, *p* < 0.001). The likelihood of psychiatrists supporting the biological concept is more than three times that of theologians (OR = −3.18, β = −0.320, *p* = 0.001), while their support for the social constructivist concept is more than twice as likely (OR = −2.65, β = −0.360, *p* = 0.008).

**Figure 2:**
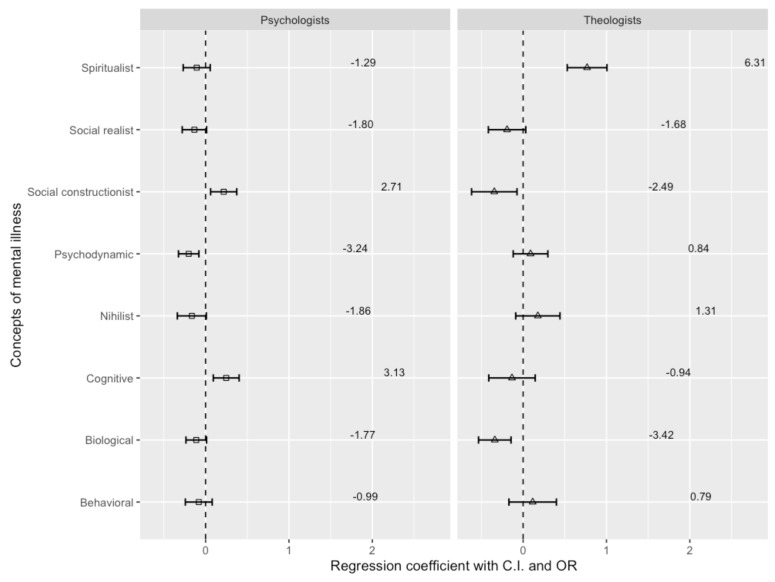
Psychiatrists’, psychologists’, and theologians’ concepts of schizophrenia.

Figure 2 shows the results of a multinomial regression analysis of implicit models for schizophrenia, which shows differences between psychiatrists as a reference group compared to psychologists and theologians. There are two models or concepts of mental disorders when it comes to schizophrenia specific to psychologists as opposed to psychiatrists. Psychologists are more than twice as likely (OR = 2.71, β = 0.217, *p* = 0.007) to use the social constructivist model as compared to psychiatrists. Also, even more, among psychologists, the probability of using a cognitive model in schizophrenia is more than three times higher (OR = 3.13, β = 0.247, *p* = 0.002). On the other hand, psychiatrists use the psychodynamic model to explain schizophrenia with a probability higher than three times (OR = 3.24) that of psychologists. Also, we can expect a biological (OR = 1.77), nihilistic (OR = 1.86), and socially realistic (OR = 1.80) model of considering schizophrenia with a higher probability in psychiatrists than in psychologists. Psychologists have a lower preference for biological, social realism, and nihilistic models compared to psychiatrists, but these differences are not statistically significant.

Among theologians, compared to psychiatrists, the spiritual model dominates, where there is a more than six times higher probability (OR = 6.313, β = 0.768, *p* < 0.001) of a spiritual explanation of the etiologyand treatment of schizophrenia. Conversely, psychiatrists, in contrast to psychologists, show more than three times the probability of using the biological model (OR = 3.42) and more than two times the probability (OR = 2.49) of using the social constructivist model.

**Figure 3:**
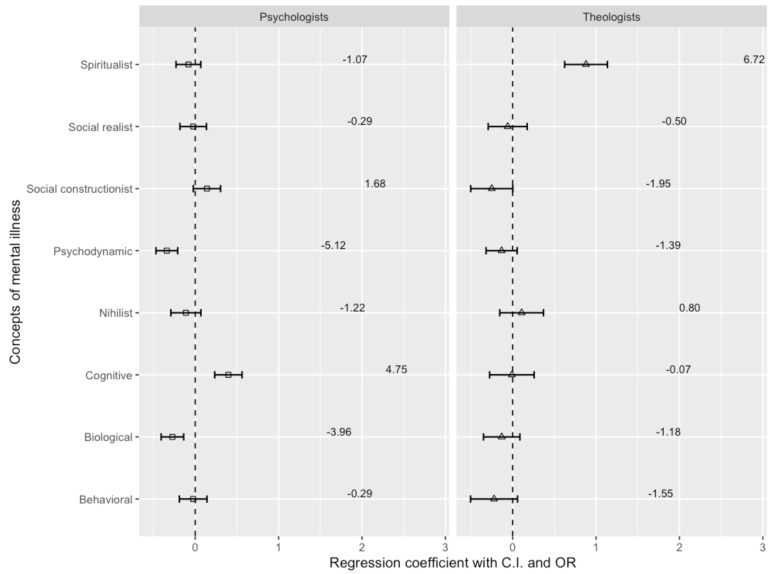
Psychiatrists’, psychologists’, and theologians’ concepts of generalized anxiety disorder.

Figure 3 shows the results of a multinomial regression analysis of implicit models for generalized anxiety disorder, which shows differences between psychiatrists as a reference group compared to psychologists and theologians.

There are three conceptions regarding generalized anxiety disorder that psychiatrists and psychologists differ on. Psychiatrists prefer the psychodynamic and biological models, whereas psychologists favor the cognitive idea. Psychiatrists are almost four times more likely to advocate the biological (OR = −3.96, β = −0.273, *p* < 0.001) and five times more likely to accept the psychodynamic (OR = −5.12, β = −0.340, *p* < 0.001) model. Psychologists are almost five times more likely than psychiatrists to accept the cognitive concept (OR = 4.75, β = 0.398, *p* < 0.001) of generalized anxiety disorder.

Theologians significantly prefer the spiritual concept of generalized anxiety disorder and are six times more likely than psychiatrists to prefer the spiritual concept (OR = 6.72, β = 0.879, *p* < 0.001). There is no significant difference in other concepts.

**Figure 4:**
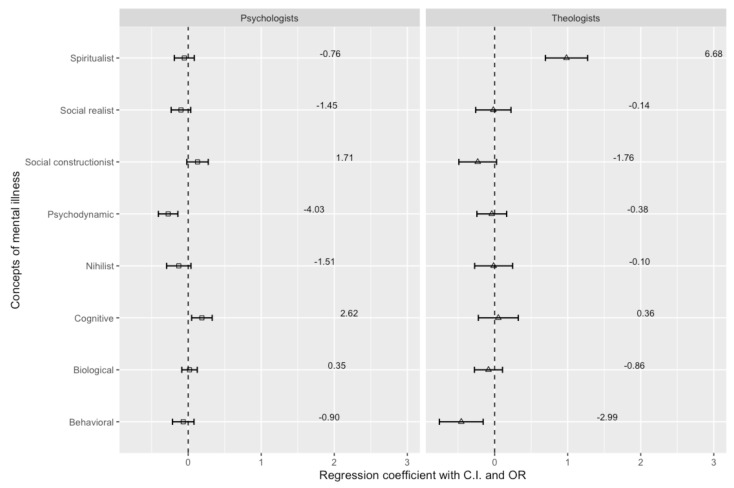
Psychiatrists’, psychologists’, and theologians’ concepts of antisocial personality disorder.

Figure 4 shows the differences in the probability of choosing a particular concept of antisocial personality disorder.

Psychiatrists and psychologists differ in their preference for the concept of antisocial personality disorder. Psychiatrists are four times more likely than psychologists to advocate the psychodynamic model (OR = 4.03, β = −0.274, *p* < 0.001). Psychologists compared to psychiatrists are more than twice as likely to prefer the cognitive model (OR = 2.62, β = 0.188, *p* = 0.009).

Theologians, in comparison to psychiatrists, advocate a behavioral and spiritual model to a greater extent. Theologians will be six times more likely to choose a spiritual concept than psychiatrists. There is the same relationship with the behavioral concept; the probability of choosing the specified model among theologians is three times higher than among psychiatrists (OR = 2.99, β = 0.984, *p* < 0.001).

## 4. Discussion

The results demonstrate contrasting perspectives on mental disorders between psychiatrists, psychologists, and theologians. These differences are not the same in all mental disorders. Different mental disorders have varying concepts and perceptions. This research holds substantial weight and importance due to its simultaneous examination of three samples, namely psychologists, psychiatrists, and theologians.

The descriptive statistics of attitude scores by model and disorder have similarities and differences when compared to previous research. The Harland et al. [39] research aligns with the finding that psychiatrists exhibit the highest average values for the biological concept in schizophrenia and depression. The research published by Harland et al. [39] supports the idea of a less pronounced biological model in generalized anxiety and antisocial personality disorder. The results indicate that psychodynamic and behavioral concepts are given more significance concerning anxiety and personality disorders.

Psychologists’ descriptive statistics outcomes contrast with those reported in the research by Read et al. [41] in the UK. Psychologists exhibit the highest average scores in schizophrenia and depression across biological, cognitive, and behavioral domains, while the Read et al. [41] study on British psychologists reveals the highest average scores in social realism and social constructivism. The above comparisons reveal contrasting concepts in mental health between Croatia and Great Britain. Education might be a contributing factor, along with cultural variations. Great Britain stands out from Croatia with its rich cultural diversity and social stratification.

The spiritual concept consistently has the highest average value among theologians compared to psychiatrists and psychologists across all four diagnoses. Nevertheless, their scores on other concepts are also notably high, matching those of other mental health professionals.

Due to limitations in interpretation and comparison, as well as the asymmetry of distributions, multinomial regression analysis was utilized to enable comparisons of mental disorder concept profiles. These statistical analyses enhance the accuracy of our results.

Psychiatrists and psychologists have varying preferences when it comes to their beliefs about mental disorders, such as biological, cognitive, social constructivist, psychodynamic, and spiritual concepts. In understanding schizophrenia, psychiatrists are more likely to embrace the psychodynamic concept, whereas psychologists are more inclined towards the cognitive and social constructivist concept. In dealing with depression, psychiatrists are more likely to consider biological, psychodynamic, and spiritual concepts, while psychologists tend to favor the cognitive concept. Interestingly, psychologists are the least spiritually inclined among psychiatrists and theologians, regardless of mental disorder.

The psychodynamic and biological concept is favored by psychiatrists for generalized anxiety disorder, whereas psychologists prefer the cognitive concept. When discussing antisocial personality disorder, psychiatrists lean towards the psychodynamic concept, while psychologists favor the cognitive concept.

It was expected that psychiatrists, psychologists, and theologians would exhibit differences in their preference for the biological model. Psychiatrists and psychologists differ in their preference for the psychodynamic model, with psychiatrists consistently favoring it. Psychiatrists and psychologists are indistinguishable in the biological concept, such as when considering schizophrenia. The difference becomes evident when comparing them to theologians. As previously mentioned, psychologists strongly support the biological concept with high results.

The main limitation of this study lies in its methodology, specifically the use of online questionnaire surveys. By conducting it this way, we cannot control how respondents fill out the questionnaire, nor can we gather their additional reactions. Furthermore, we employed the MAQ questionnaire, but it is just one of many methods, making it uncertain how different results would be with another tool and approach.

Cross-sectional research involving psychologists, psychiatrists, and theologians from diverse countries and cultures should be conducted in the future. Additionally, a more comprehensive research methodology, like qualitative methods such as in-depth interviews or group discussions, should be employed. Including laypeople in this research would be valuable due to the increasing prevalence of mental disorders as a public health concern in the 21st century. The lack of consensus regarding the definition, causes, classification, and treatment of mental health highlights the need to prioritize theoretical work alongside research.

## 5. Conclusions

The prevalence of mental disorders is becoming a major global challenge, impacting health, economy, and society. Effective and comprehensive systemic solutions are needed to address the ongoing prevalence of mental disorders. Various theoretical models explain the causes and mechanisms of their occurrence, which it is important to comprehend. Mental disorders are the product of a complex interplay of biological, psychological, social, and spiritual factors, as described by the biopsychosocial–spiritual model. Research is uncovering the relationship between religion, spirituality, and mental well-being, stressing the significance of cooperation between religious representatives and mental health professionals. Evidence shows that such collaboration brings benefits like increased referrals to mental health specialists and reduced stigma around mental illness.

Research shows that psychiatrists, psychologists, and theologians have distinct perspectives on mental disorders, with pros and cons for each model. One of the beneficial differences is the potential for diverse, holistic treatment methods. Psychiatrists emphasize biological models, psychologists focus on cognitive and social constructivist models, and theologians consider spiritual aspects, each discipline providing distinct insights for personalized treatment approaches. Nevertheless, harmful variations can result in misunderstandings, conflicts within interdisciplinary teams, and fragmented care, ultimately perplexing patients and diminishing treatment quality. To handle this situation, it is advisable to implement joint training which will homogenize knowledge, encourage collaboration, and build integrated treatment models that combine the strengths of different disciplines, ultimately enhancing mental healthcare outcomes.

To fully comprehend mental disorders, future studies must adopt a multidisciplinary approach, involving psychologists, psychiatrists, and theologians from diverse countries and cultures. By integrating various perspectives and cultural insights, we can enhance our comprehension of mental health. Given the complexity of these issues, collaboration across disciplines is essential, as each field offers unique expertise to enhance interventions and policies.

Comprehensive research methodologies, including qualitative methods like in-depth interviews and focus groups, are crucial. These techniques allow for the gathering of precise information that captures the real-life experiences of people dealing with mental health issues, leading to a more profound comprehension of the circumstances and significance of their difficulties. Participants can share their stories through qualitative approaches, unveiling common themes and cultural differences that enhance the research narrative and inform treatment strategies.

Another crucial aspect is including laypeople in research. Given the growing urgency of mental disorders as a public health issue, it is vital to actively involve those who are affected. Lay perspectives offer valuable insights into mental health, helping researchers understand real-world implications and tailor interventions.

The absence of agreement on mental health definitions, causes, classification, and treatment emphasizes the necessity for theoretical work and empirical research. The field’s progress relies on establishing a clear framework to understand mental health. This requires evaluating established theories, incorporating novel discoveries, and encouraging conversations that merge various viewpoints. By focusing on theoretical work, researchers can build a strong foundation for future studies, enhancing mental health research quality and advancing a holistic approach to care for individuals with mental health challenges. 

## Figures and Tables

**Table 1:** Sample distribution by demographic variables.

Characteristic	Psychiatrists, N = 122	Psychologists, N = 116	Theologists, N = 74	*p*-Value
Sex				<0.001
Male	44 (36%)	10 (8.6%)	74 (100%)	
Female	78 (64%)	106 (91%)	0 (0%)	
Age				
−25	0 (0%)	2 (1.7%)	0 (0%)	
26–35	23 (19%)	52 (45%)	18 (24%)	
36–45	34 (28%)	31 (27%)	22 (30%)	
46–55	44 (36%)	24 (21%)	11 (15%)	
56–65	21 (17%)	7 (6.0%)	23 (31%)	
Religion				
Atheist	6 (4.9%)	26 (22%)	0 (0%)	
Agnostic	15 (12%)	17 (15%)	0 (0%)	
Catholic	97 (80%)	65 (56%)	74 (100%)	
Christian	2 (1.6%)	4 (3.4%)	0 (0%)	
Islam	0 (0%)	0 (0%)	0 (0%)	
Judaism	0 (0%)	0 (0%)	0 (0%)	
Other	2 (1.6%)	4 (3.4%)	0 (0%)	

**Table 2:** Descriptive statistics for the aggregate attitude scores by model and disorder among psychiatrists, psychologists, theologians.

Concepts	Psychiatrists	Psychologists	Theologians
Schizophrenia			
Biological	15.3 (2.25)	15.1 (2.64)	12.8 (2.4)
Cognitive	9.7 (2.65)	9.9 (2.86)	11.1 (2.42)
Behavioral	11.2 (2.56)	10.6 (2.28)	12.3 (1.9)
Psychodynamic	11.2 (2.9)	9.8 (3.13)	12.8 (2.82)
Social realist	9.9 (2.74)	9.1 (2.77)	11.7 (2.46)
Social constructionist	7.7 (2.39)	7.8 (2.86)	9.3 (2.63)
Nihilist	6.8 (1.89)	6.3 (2.22)	8.6 (2.28)
Spiritualist	7.1 (2.4)	6.3 (2.63)	11.6 (2.41)
Major depressive disorder			
Biological	14.7 (2.27)	13.8 (2.69)	12.6 (2.21)
Cognitive	11.2 (2.76)	13.1 (2.96)	11.9 (2.25)
Behavioral	12.1 (2.61)	12.8 (2.40)	12.6 (1.81)
Psychodynamic	12.2 (3.00)	11.1 (3.08)	13.2 (2.33)
Social realist	11.4 (2.90)	11.6 (2.70)	12.7 (2.35)
Social constructionist	8.1 (2.46)	8.3 (2.92)	9.6 (2.57)
Nihilist	6.9 (2.01)	6.5 (2.24)	8.6 (2.35)
Spiritualist	7.8 (2.64)	7.1 (2.90)	12.5 (2.15)
Generalized anxiety disorder			
Biological	14 (2.55)	12.7 (2.57)	12.6 (2.16)
Cognitive	11.7 (2.71)	13.6 (2.85)	11.8 (2.15)
Behavioral	12.7 (2.63)	13.2 (2.40)	12.7 (1.75)
Psychodynamic	13.2 (2.74)	11.2 (3.15)	13.0 (2.50)
Social realist	11.9 (2.50)	11.9 (2.42)	12.9 (2.38)
Social constructionist	8.2 (2.46)	8.5 (2.92)	9.6 (2.52)
Nihilist	7.2 (2.02)	6.7 (2.41)	8.7 (2.36)
Spiritualist	7.9 (2.60)	7.1 (3.04)	12.6 (2.20)
Antisocial personality disorder			
Biological	12.7 (2.86)	12.9 (2.54)	12.4 (2.35)
Cognitive	11.8 (2.62)	11.7 (2.73)	11.9 (2.08)
Behavioral	13.2 (2.57)	12.3 (2.45)	12.7 (1.81)
Psychodynamic	13.3 (2.66)	11.2 (3.11)	13.2 (2.24)
Social realist	12.1 (2.47)	10.9 (2.78)	13.1 (2.29)
Social constructionist	8.7 (2.47)	8.3 (2.84)	9.8 (2.52)
Nihilist	7.6 (2.14)	6.7 (2.36)	8.6 (2.37)
Spiritualist	8.1 (2.61)	7.0 (2.79)	12.6 (2.21)

## Data Availability

Data are available in principal research zkralj@kbsplit.hr.
